# Spatial distribution of *Anopheles gambiae* sensu lato larvae in the urban environment of Yaoundé, Cameroon

**DOI:** 10.1186/s40249-019-0597-6

**Published:** 2019-10-09

**Authors:** Landre Djamouko-Djonkam, Souleman Mounchili-Ndam, Nelly Kala-Chouakeu, Stella Mariette Nana-Ndjangwo, Edmond Kopya, Nadége Sonhafouo-Chiana, Abdou Talipouo, Carmene Sandra Ngadjeu, Patricia Doumbe-Belisse, Roland Bamou, Jean Claude Toto, Timoléon Tchuinkam, Charles Sinclair Wondji, Christophe Antonio-Nkondjio

**Affiliations:** 1 Malaria Research Laboratory, Organization for the fight against Endemic diseases in Central Africa (OCEAC), P.O. Box 288, Yaoundé, Cameroon; 2 Vector Borne Infectious Disease Unit of the Laboratory of Applied Biology and Ecology (VBID-LABEA), Department of Animal Biology, Faculty of Science, University of Dschang, P.O. Box 067, Dschang, Cameroon; 3 Faculty of Science, University of Yaounde I, P.O. Box 337, Yaounde, Cameroon; 4 Faculty of Health Sciences University of Buea, P.O. Box 63, Buea, Cameroon; 5 Vector Biology Liverpool School of Tropical medicine Pembroke Place, Liverpool, L3 5QA UK

**Keywords:** Larval habitat, Anopheles dynamics, Urban environment, Malaria, GIS, Yaoundé, Cameroon

## Abstract

**Background:**

The rapid and unplanned urbanization of African cities is considered to increase the risk of urban malaria transmission. The present study objective was to assess factors influencing the spatio-temporal distribution of *Anopheles gambiae* s.l. larvae in the city of Yaoundé, Cameroon.

**Methods:**

All water bodies were checked once every 2 months for the presence of mosquito larvae from March 2017 to May 2018 in 32 districts of Yaoundé. Physico-chemical characteristics including the size, depth, turbidity, pH, temperature, conductivity, sulfates, organophosphates, hydrogen peroxide (H_2_O_2_), conductivity, iron and calcium were recorded and analyzed according to anopheline larvae presence or absence. High resolution satellite images from landsat sentinel Enhanced Thematic Mapper were used for spatial mapping of both field and environmental variables. Bivariate and multivariate logistic regression models were used to identify variables closely associated with anopheline larvae distribution.

**Results:**

A total of 18 696 aquatic habitats were checked and only 2942 sites (15.7%) contained anopheline larvae. A high number of sites with anopheline larvae (≥ 69%) presented late instar larvae (L3, L4 and pupae). Anopheline mosquito larvae were sampled from a variety of breeding sites including puddles (51.6%), tire prints (12.9%), wells (11.7%) and drains (11.3%). Bivariate logistic regression analyses associated anopheline larvae presence with the absence of predators, absence of algae, absence of vegetation and depth of less than 1 m. Conductivity, turbidity, organophosphates, H_2_O_2_ and temperature were significantly high in breeding sites with anopheline larvae than in breeding sites without these larvae (*P* <  0.1). Anopheline species collected included *An. coluzzii* (91.1%) and *An. gambiae* s.s. (8.9%). GIS mapping indicated a heterogeneous distribution of anopheline breeding habitats in the city of Yaoundé. Land cover analysis indicated high variability of the city of Yaoundé’s landscape.

**Conclusions:**

The data confirms adaptation of *An. gambiae* s.l. to the urban domain in the city of Yaoundé and calls for urgent actions to improve malaria vector control.

## Multilingual abstracts

Please see Additional file 1 for translations of the abstract into the five official working languages of the United Nations

## Background

The rapid unplanned urbanization of sub-Saharan Africa cities, is significantly affecting the epidemiology of vector borne diseases [1–8]. During the last decade, several outbreaks of chikungunya and dengue have been reported in major cities across sub-Saharan Africa [9–12]. For malaria, although successful scale up of control tools such as long lasting insecticidal nets (LLINs) and indoor residual spraying permitted to reduce the global burden of the disease across the continent [13], the disease is still highly prevalent in most urban settings [2, 14–17]. The fast growing urban population is considered to exert a high pressure on land resources resulting in the colonization of marshland and swamps and expansion of deforestation. The said conditions occurring at a large scale, create suitable breeding opportunities for mosquitoes increasing by then, malaria transmission risk in urban settings. Understanding anopheline larvae dynamic in this changing environment becomes imperative for the implementation of successful control measures.

In Cameroon, malaria is highly endemic and the whole country is exposed to the risk of transmission [18]. Although increased malaria transmission has been reported in urban settings [14], there is still not enough data on the spatio-temporal distribution of anopheline population breeding habitats. The characterization of anopheline breeding sites in the cities of Douala and Yaoundé indicated that anopheline larvae could colonize polluted breeding sites and that this adaptation could affect the susceptibility level of adult mosquito populations to insecticides [19]. The study by Kamdem et al. [20] along a transect between rural and urban settings, suggested that ecological correlates and some environmental indicators, could be good predictors for discriminating the distribution of *An. gambiae* sensu stricto and *An. coluzzii* in the city of Yaoundé [20]. However, the study did not provid detailed information on the spatio-temporal distribution of anopheline breeding habitats in the city. Indeed, information on the spatial distribution of mosquito breeding habitats is required to enable tracking and targeting hotspot areas where there is persistent presence of vector populations. The development of geographic information systems (GIS) mapping technology now provides an opportunity to have affordable and scalable approaches that deliver high resolution maps of mosquito distribution or transmission risk which could enable better surveillance system [21].

In Cameroon, insecticide-treated nets are the main measures used for controlling mosquito populations but this measure is affected by the rapid emergence of insecticide resistance particularly in urban settings [22, 23]. In this context, a deep understanding of the influence of ecological factors and spatio-temporal distribution of mosquitoes could enable the implementation of new interventions such as larval control to improve the performance of interventions implemented in the field. Compared to East Africa, there has been little effort in using larval source management for controlling vector-borne diseases in Central Africa [24–27]. Larval control has proved to be efficient in situation where habitats are few, fix and manageable [28–32]. In most sub-Saharan Africa cities, malaria transmission is focal and depends on the existence of hotspot areas [2, 27, 33–35]. Identifying these hotspot areas and mapping the distribution of anopheline species across the year could be key for the implementation of a successful and sustainable program for malaria control and elimination in urban settings.

The aim of the present study was to assess the spatio-temporal distribution of aquatic stages of anopheline mosquitoes in Yaoundé and the influence of physico-chemical parameters and the landscape on anopheline species distribution.

## Methods

### Study area

The study was conducted in Yaoundé (3°43′00″ to 3°58′00″ N and 11°24′30″ to 11°34′30″ E), the capital city of Cameroon. Yaoundé is a town covering a surface area of 304 km^2^ of about 3 million inhabitants situated between 700 and 750 m above sea level. The city is drained by several permanent streams and is situated within the Congo-Guinean phytogeographic zone [36]. The climate is of equatorial type with four seasons (two rainy seasons [September–November and March–June], two dry seasons [December–February and July–August]) [37]. The average rainfall in Yaoundé is estimated at 1688 mm/year, thaverage annual temperature is of 26.31 °C varying between 16 to 33 °C depending on the season. The average humidity is 80% and varies during the day between 35 to 98% [38]. The city is exposed to frequent humid winds blowing south-west to west or north to west [39].

### Spatial and temporal mapping of water bodies using the geographic information system (GIS)

High resolution satellite images from landsat sentinel Enhanced Thematic Mapper (ETM) at the resolution of 15 m were acquired for the city of Yaoundé. Images used were from two distinct periods May 2017 and June 2018. The satellite image includes four spectral bands green, red, blue and near-infrared. The image was projected to Universal Transverse Mercator (UTM) zone 32 on the World Geodetic System (WGS) 84 Datum. The image was analyzed using the software Erdas Imagine 2014 (Géomatica-SIG and télédetection-Intergraph, US). A supervised classification was applied. A training dataset was selected on the satellite image manually by digitizing multiple training polygons for each class. One hundred training sites were obtained for each of the land cover as done elsewhere [40]. Data recorded for spatial mapping included both field and environmental variables. On the field the geographical coordinates of all breeding habitats were registered using a Garmin-branded GPS (Garming eTrex 10 Handheld). Data extraction was done using Mapsource software. Once collected, field data were crosschecked and link to GPS coordinates for each district with a unique code for each data. All the geo-databases were then registered in ArcGIS 10.2.2 software (ESRI, Redland, CA, USA). Arc-catalog was used to construct the Geo-database that enabled visualization of the spatial distribution of breeding sites. Environmental variables including humidity, vegetation and soil indicators were calculated from the landsat sentinel 2 images. The Global Moran statistics index in ArcGIS was used to test for spatial autocorrelation [41]. The Normalised Difference Vegetation Index (NDVI) was calculated using the near infrared (NIR) and red spectral bands as (NIR-Blue)/(NIR + Blue). The Normalized Difference Water Index (NDWI) was calculated as (NIR-Green)/(NIR + Green) [40]. Building Index (BI) calculations were done as follows BI = sqr (NIR^2^ + Red^2^). Calculations were done using Erdas Imagine 2014.

### Breeding sites sampling

**Fig. 1:**
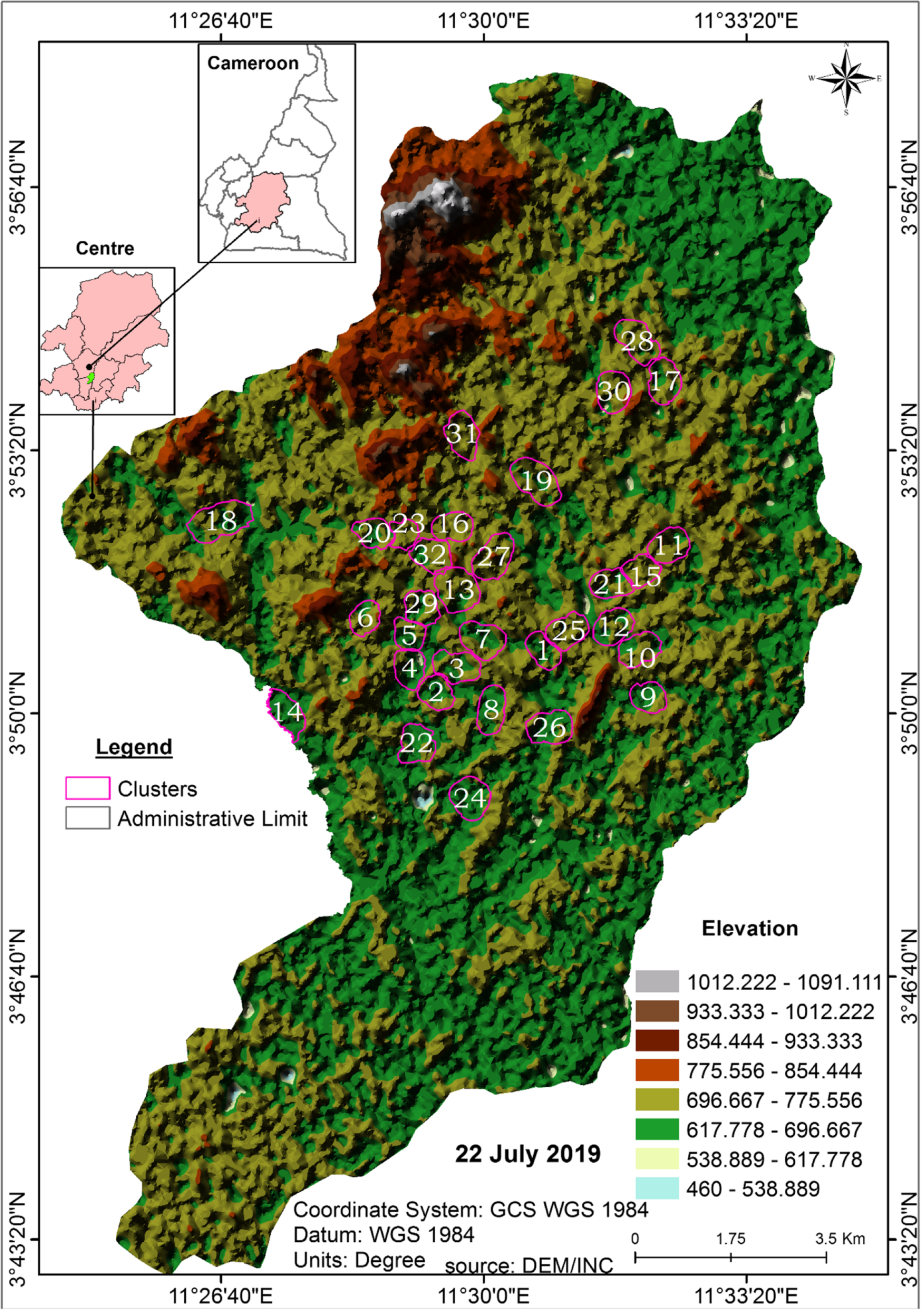
A digital elevation model (DEM) image of the city of Yaoundé showing elevation of study sites

Mosquito breeding sites prospections were conducted in 32 districts out of about 100 in Yaoundé from March 2017 to May 2018 once every 2 months. The districts were characterized by an alternation of highland and lowland areas and extended from the city centre to the periphery (Fig. 1). Most of the lowland areas are irrigated by the river system in the city of Yaoundé including Mfoundi, Biyeme and Mefou rivers, whose edges provide excellent breeding opportunities for mosquitoes during the dry season. The districts are highly populated with constructions in both highland and lowland areas. Marshlands along Mefou River are exploited for house construction and for the practice of market gardening during the dry season. These areas are the main sources of breeding habitats for mosquitoes. All water bodies encountered were geo-located using a Garmin eTrex® GPS and enter to a GIS database for analysis.

### Physico-chemical characterization of breeding sites larval collection and mosquito species identification

Parameters recorded in each breeding site included breeding habitats type, size, depth, exposure to sunlight, presence of vegetation, the distance between each water body and the nearest human dwellings and the presence/absence of predators, the status organically polluted or not, the proportion of water surface covered by vegetation or algae, breeding sites type (stagnant water pools, gutters, well, tire print, footprint, pit latrine ….). In addition to these, the following parameters were also recorded Total Dissolved Solids (TDS) pH, temperature, conductivity using a Jenway multiparametric probe. The concentrations of sulfates, organophosphates, hydrogen peroxide (H_2_O_2_), turbidity, iron and calcium were analyzed using a Wagtech spectrophotometer [42].

All water bodies were checked for the presence of mosquito larvae. The immature stages of mosquitoes were collected using the standard dipping technique [43]. Three to five dips using a deeper of 350 ml were performed for small breeding sites of less than 1 m^2^; and 5 to 10 dips were undertaken in breeding sites of more than 1 m^2^. For some habitats such as tire print or foot print which could be too shallow at some periods, larval collection was conducted using a pipette. The average larval density (N) was estimated as the ratio of the number larvae collected per dip (using a dipper with a volume of 350 ml). Once collected larvae were classified according to their stages: early instars larvae (L1 and L2) and late instars (L3, L4 and pupa). Anopheline larvae were separated from the culicines using morphologically identification keys [44, 45]. Each anopheline larvae specimen was stored individually at − 20 °C.

Mosquitoes larvae belonging to the *An. gambiae* complex were further processed by PCR [46], to distinguish *An. coluzzii* from *An. gambiae* s.s*.* the two members of the complex found in Yaoundé. DNA extracted from larvae according to Livak protocol [47], were used for these analyses.

### Data analysis

MedCalc statistical software version 15.8 (Medcalc Software bvba, Ostend Belgium; https://www.medcalc.org; 2015) was used to calculate the odd ratio and 95% confidence interval to assess the influence of various environmental variables on anopheline larval presence. Student test was performed to compare the physico-chemical characteristics between water bodies with and without anopheline larvae. Bivariate and multivariate logistic regression models were used to identify variable associated with presence of anopheline larvae. The significance level was fixed at α < 0.05. A linear discriminant function analysis was used to determine key factors associated with *An. gambiae* s.s. and *Anopheles coluzzii* larvae presence. Analyses were performed using SPSS Statistics for Windows, version 20 (SPSS Inc., Chicago, Ill., USA) and the R software version 3.5.2 (R Core Team (2018) R Foundation for Statistical Computing, Vienna, Austria URL https://www.R-project.org/).

## Results

### Anopheline larvae distribution

A total of 18 696 aquatic habitats were prospected and 2942 (about 15.7%) were found with anopheline larvae. The proportion of sites found with anopheline larvae varied significantly according to districts (*P* <  0.05). High anopheline larval densities (> 50 larvae/breeding site) were recorded in 56.9% (1674/2942) of positive breeding sites and late instar larvae (L3, L4, pupae) were recorded in 69.3% (*n* = 2038) habitats.

Anopheline larvae were found in a variety of breeding habitats. Puddles were the most common representing 51.6% of the total anopheline breeding sites. Tire prints, wells and drains represented 12.9, 11.7 and 11.3% respectively of breeding sites with anopheline larvae. The remaining breeding habitats (12.5%) included containers (buckets and tires), foot prints, pits and river edges (Table 1). Most breeding sites with anopheline larvae had less than 1 m^2^ of surface. In almost all breeding sites, anopheline and culicine larvae were found in sympatry.

**Table 1:** Characteristics of the different types of breeding habitats recorded in the city of Yaoundé

Types of sites	Number of sites (%)	% with anopheline larvae	% with size < 1 m	% > 25 vegetation coverage	% with algae	% with culicine larvae
Puddles	273 (51.6)	50.5	44.3	17.2	17.9	22
Tire prints	68 (12.9)	67.6	58.8	2.9	7.3	22.1
Foot prints	11 (2.1)	91	81	10	0	41
Wells	62 (11.7)	17.7	51.6	11.3	30.6	11.3
Drains	60 (11.3)	25	41.7	36.7	45	21.7
Furrows	44 (8.3)	29.5	15.9	36.4	18.2	29.5
Artificial containers^a^	4 (0.8)	25	100	0	25	0
Others^b^	7 (1.3)	14.3	0	28.6	14.3	42.9

^a^Tyres and buckets; ^b^Ponds, pits and Rivers edges

### Logistic regression analysis between anopheline larval presence and breeding habitats characteristics

Bivariate logistic regression analyses were conducted to assess any association between anopheline larvae presence and some breeding sites characteristics. It appears that anopheline larvae were particularly frequent in the absence of predators (larvivorous fishes) (*OR* = 3.4, *P* <  0.0001), absence of algae (*OR* = 1.5, *P* = 0.06), and in breeding sites situated less than 10 m from living habitations (*OR* = 1.7, *P* = 0.002) (Table 2).

**Table 2:** Characterization of anopheline larvae breeding sites

Characteristics	Variables	Number of BS sampled	Number of BS with anopheline larvae (%)	Odd ratio (95% *CI*)	*P*-value
Proximity to living habitats (meter)	≤ 10	283	108 (38.2)	1	0.002
	> 10	247	127 (51.4)	1.7 (1.2–2.4)	
Size (m^2^)	≤ 1	228	138 (60.5)	1	< 0.0001
	> 1	302	97 (32.1)	0.3 (0.2–0.4)	
Depth (meters)	< 1	465	223 (48.0)	1	< 0.0001
	≥ 1	65	12 (18.5)	0.25 (0.1–0.5)	
Permanency	Temporal	397	207 (52.1)	1	< 0.0001
	Semi-permanent	64	16 (25.0)	0.3 (0.2–0.5)	
	Permanent	69	12 (17.4)	0.2 (0.1–0.4)	< 0.0001
Vegetation cover (%)	0–25	439	213 (48.5)	1	< 0.0001
	25–75	65	13 (20.0)	0.3 (0.1–0.5)	
	75–100	26	6 (23.1)	0.3 (0.1–0.8)	0.016
Algae	Present	110	40 (36.4)	1	0.06
	Absent	420	195 (46.4)	1.5 (1.0–2.3)	
Culicine larvae	Present	115	38 (33.0)	1	0.3
	Absent	414	158 (38.2)	1.3 (0.8–1.9)	
Predators	Present	99	22 (22.2)	1	< 0.0001
	Absent	431	213 (49.4)	3.4 (0.2–5.7)	

*BS* Breeding site

### Comparison of physico-chemical characteristics of water bodies with and without anopheline larvae

A total of 11 physico-chemical parameters were measured. Three parameters including turbidity (*P* = 0.001), hydrogen peroxide (*P* = 0.002) and temperature (*P* = 0.001) were significantly high in breeding sites with *An. gambiae* s.l. larvae than in breeding sites without larvae, while conductivity indicated a border significance (*P* = 0.05) (Table 3).

**Table 3:** Comparison of physico–chemical characteristics between sites with and without anopheline larvae

Parameter	With anopheline larvae		Without anopheline larvae		*t*-test	*P*-value
	*N*	Means ± SE	*N*	Means ± SE		
pH	227	8.0 ± 0.1	286	7.8 ± 0.1	1.6	0.2
TDS (mg/L)	240	96.2 ± 16.8	300	93.9 ± 9.6	0.6	0.4
Conductivity (μs/cm)	240	449.0 ± 29.8	300	382.9 ± 14.6	3.9	0.05
Turbidity (FTU)	240	380.9 ± 103.8	300	146.0 ± 18.7	10.5	0.001
Calcium (mg/L)	240	53.7 ± 21.7	300	129.7 ± 85.6	2	0.16
Iron LR	108	0.7 ± 0.1	118	0.7 ± 0.1	0	0.98
Organophosphates (mg/L)	138	13.3 ± 4.2	158	9.0 ± 0.8	3.5	0.06
Aluminium (mg/L)	240	0.3 ± 0.1	300	0.2 ± 0.1	0.2	0.7
Sulfate	240	42.2 ± 3.9	300	36.8 ± 3.3	0.9	0.3
H_2_O_2_ (mg/L)	240	3.8 ± 1.0	300	1.1 ± 0.2	27.4	0.002
Temperature (°C)	240	28.2 ± 0.2	300	27.4 ± 0.2	1.5	0.001

*N* Number, *SE* Standard error, *FTU* Formazine turbidity unit, *TDS* Total disolved solids, *LR* Light rigid

### Seasonal dynamics of anopheline breeding habitats in Yaoundé

**Fig. 2:**
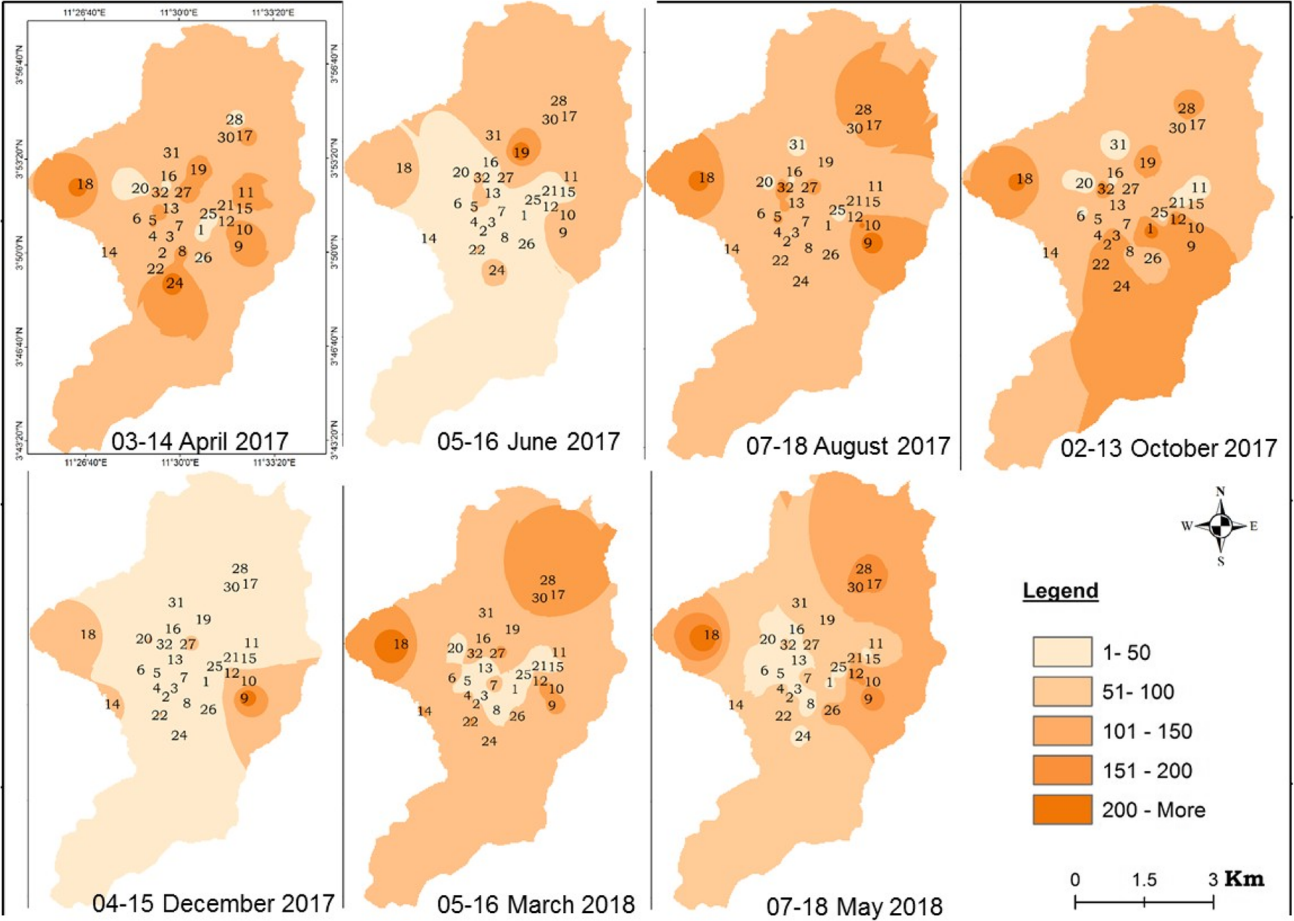
Spatial and temporal distribution of temporary breeding habitats in the city of Yaoundé

**Fig. 3:**
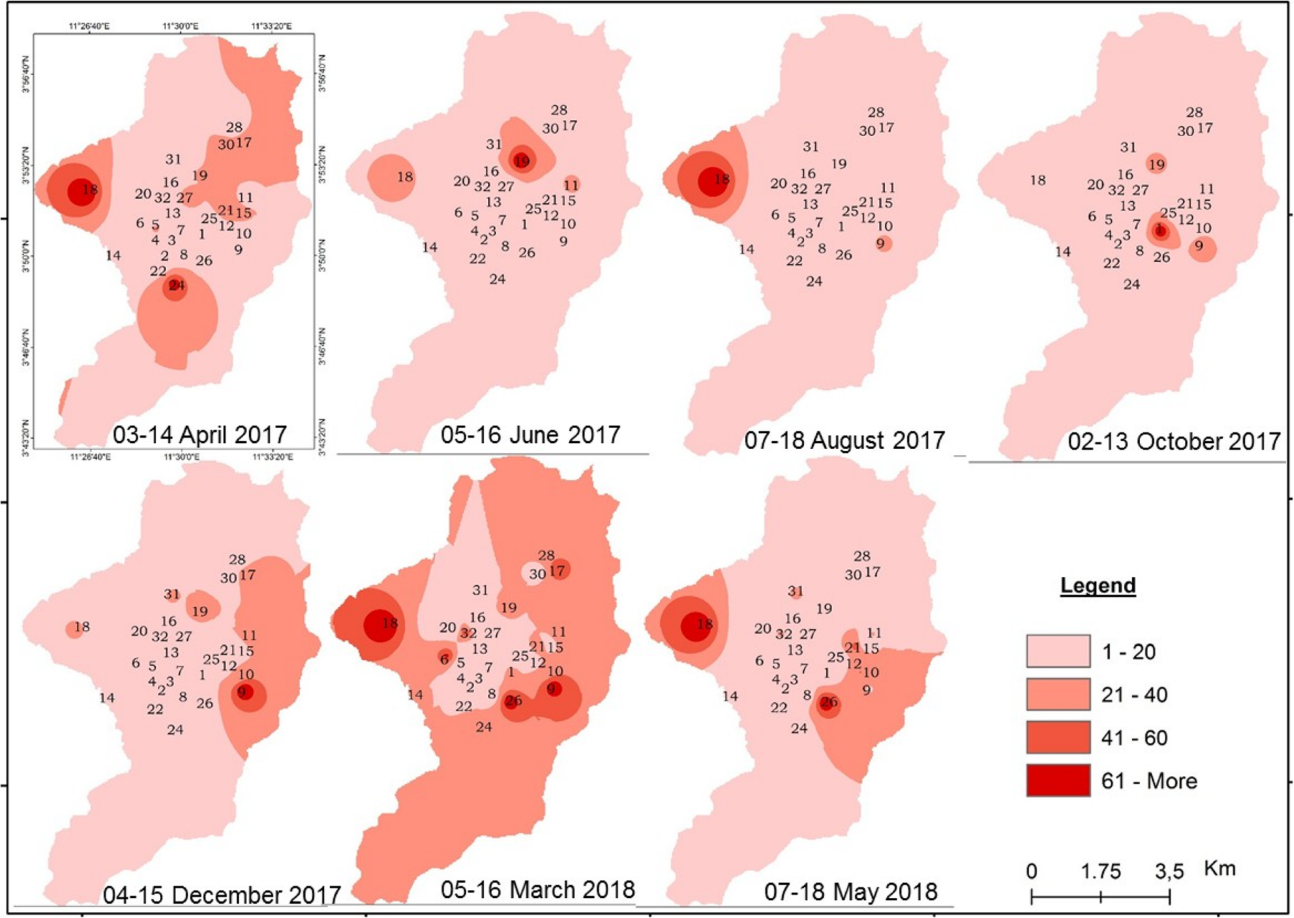
Spatial and temporal distribution of breeding sites with anopheline larvae in the city of Yaoundé

The spatial distribution of mosquitoes breeding habitats was highly variable from one season to the other. In some districts such as Nkolbisson, Ekounou-Palais, Ekounou-Ekié, Tongolo, Ngousso, Santa-Barbara and Mvog-betsi, breeding habitats were present throughout the year due to permanent water bodies such as lakes or swamps. Permanent aquatic habitats were found to maintain anopheline presence during both the dry and rainy seasons. In most districts a drastic reduction of breeding habitats was recorded during the dry season (July–August and mid-November to February) whereas the rainy season (March to June and September to mid-November) corresponded to overall increase in larval habitat count and productivity (Figs. 2 and 3). Yet, some variations were recorded during the rainy season and could result from the variability or intensity of rainfall (Fig. 3).

### Land cover and spatial mapping of mosquitoes breeding sites distribution

**Fig. 4:**
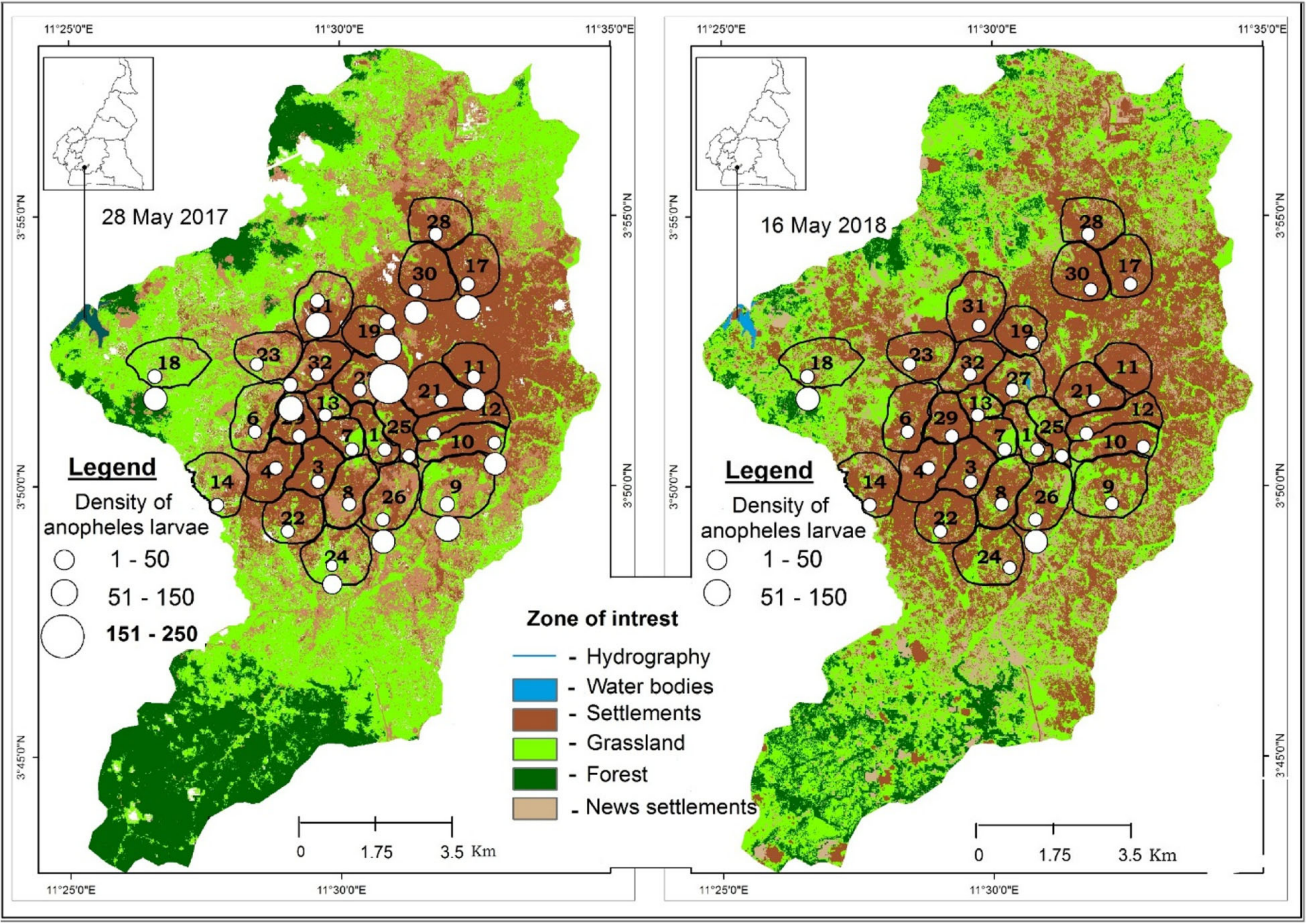
Evolution of the land cover in Yaoundé between 2017 and 2018 and the density of anopheline larvae in breeding sites

**Fig. 5:**
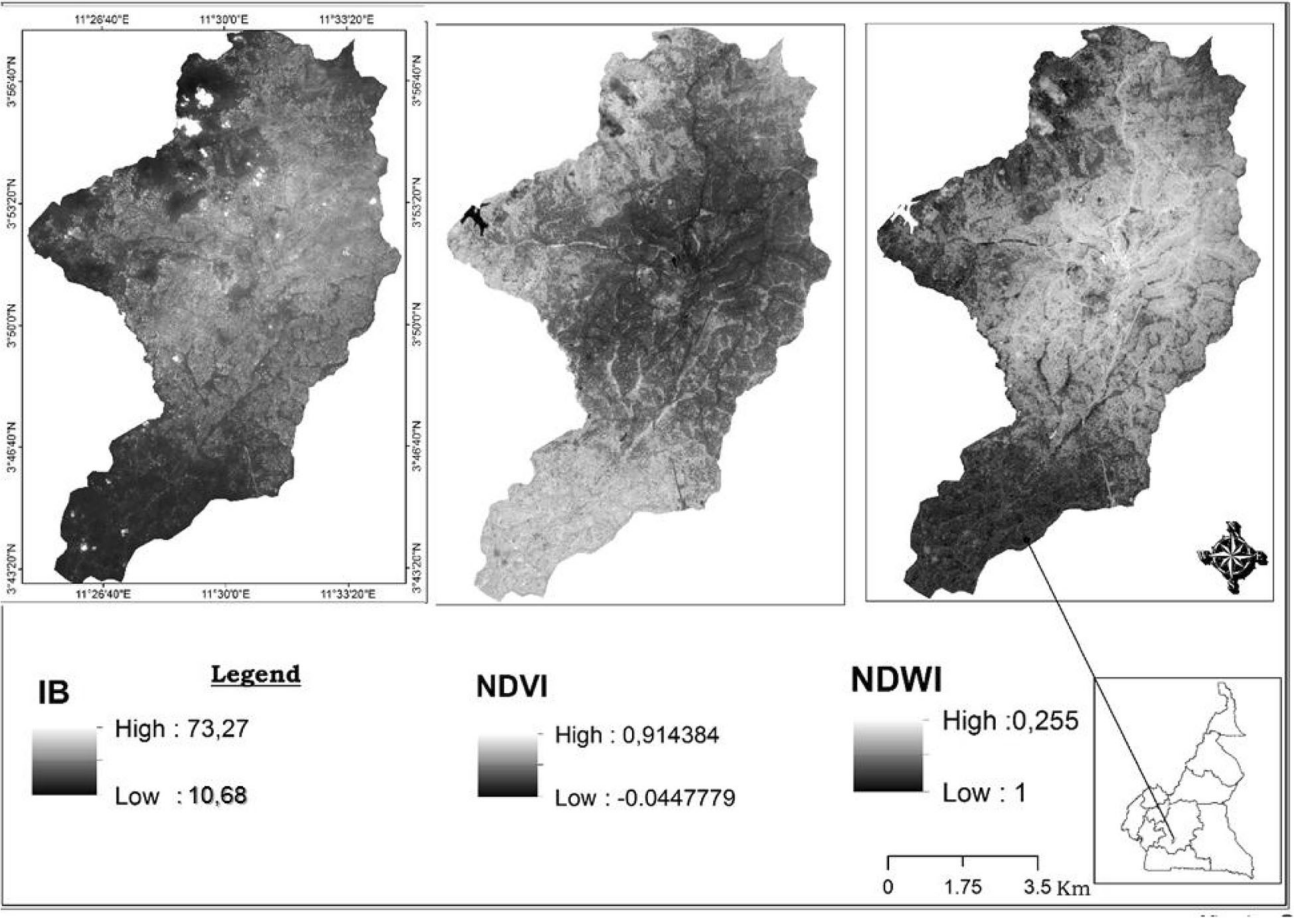
Evolution of Building Index (BI), Normalised Difference Vegetation Index (NDVI) and Normalized Difference Water Index (NDWI) in 2017 in Yaoundé

**Fig. 6:**
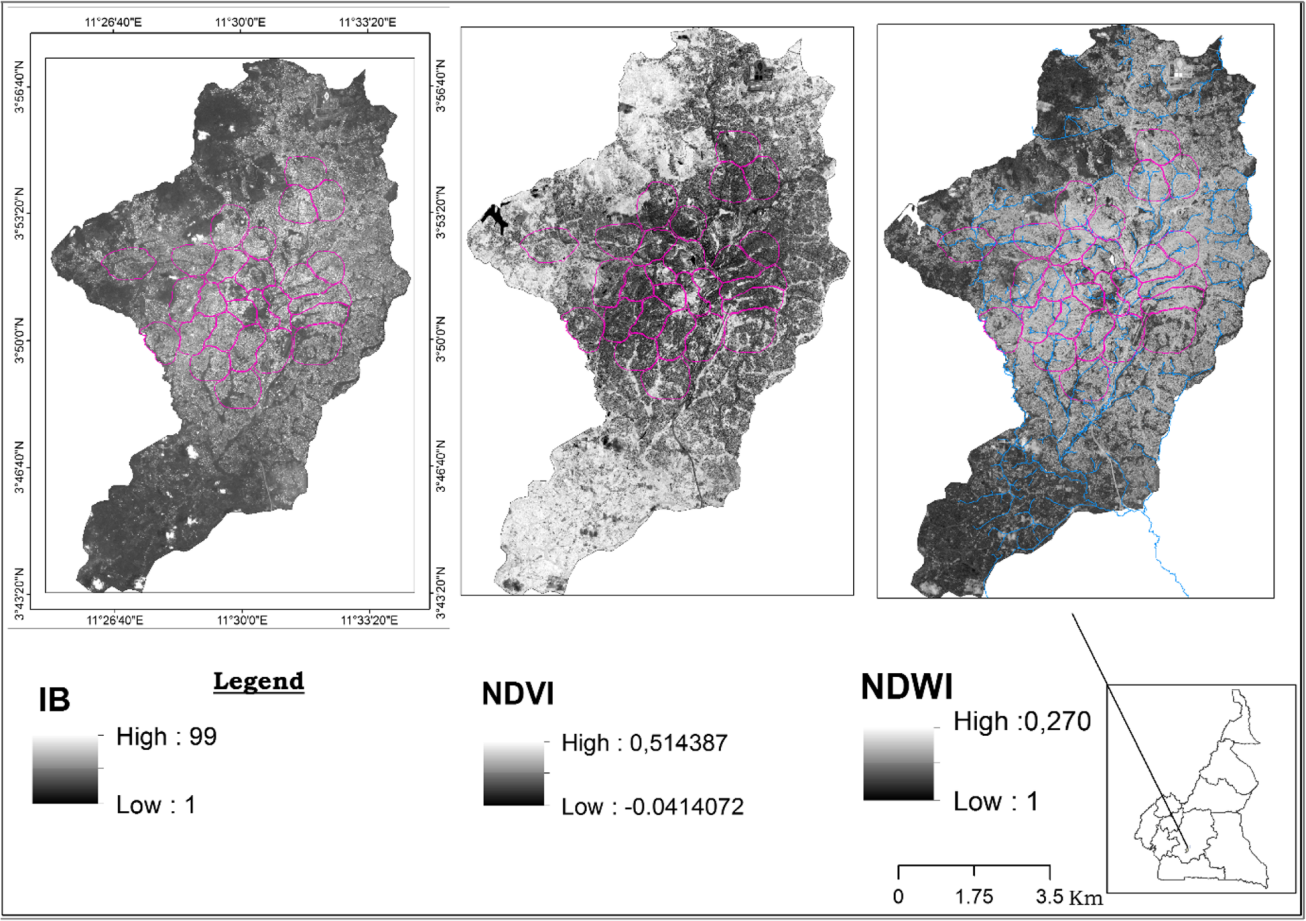
Evolution of Building Index (BI), Normalised Difference Vegetation Index (NDVI) and Normalized Difference Water Index (NDWI) in 2018 in Yaoundé

Yaoundé satellites images were examined to assess the evolution of different land cover parameters (Fig. 4) and their possible association with mosquito distribution. It clearly appeared from the images important changes in the vegetation cover and building index in and around the city between the two periods. Increased deforestation associated with increased human settlements was recorded. The forest was also replaced in different places by grassland which could be related to increase exploitation of the forest for agriculture. Four indexes were calculated to assess land use at different time periods, the Global Moran index; the Normalized Differenced Vegetation Index (NDVI), the Normalized Difference Water Index (NDWI), the Building Index (BI)*.* The Global Moran index statistic to assess the existence of any significant clustering was undertaken. No significant variation was obtained (*I* = 0.063, z-score = 1.28 and *P* = 0.2). The BI which include infrastructures such as buildings, houses, open space and road was also evaluated between the two periods calculations undertaken provided values ranging from 10.68 to 73.27 in 2017 and from 1 to 99 in 2018 (Figs. 5, 6). Variation of both the NDVI and NDWI for 2017 and 2018 are displayed in the Figs. 4 and 5.

### *An. gambiae* complex species diversity

**Fig. 7:**
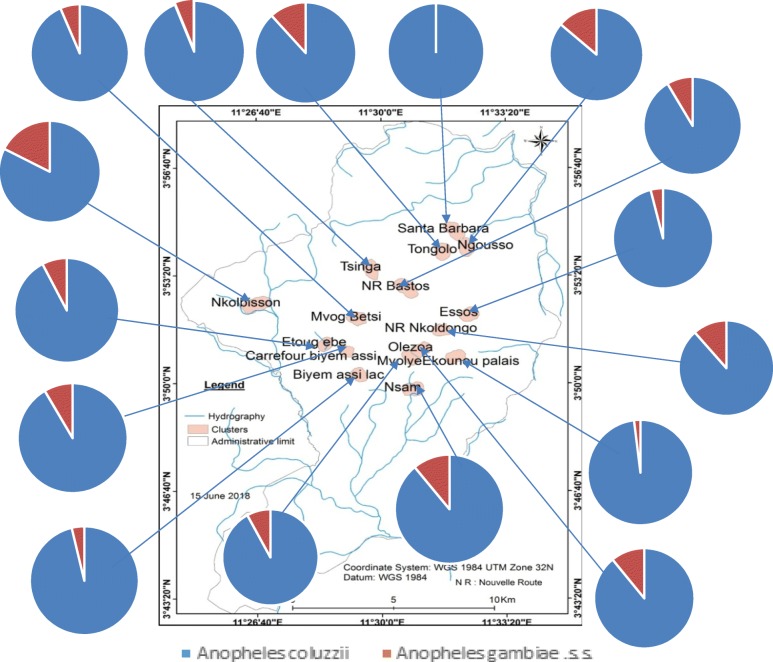
Spatial distribution of *Anopheles gambiae* and *An. coluzzii* larvae in breeding sites of some districts of Yaoundé

A total of 1237 larvae collected in 244 breeding habitats and identified morphologically as belonging to the *An. gambiae* complex were further processed molecularly to identify species of the complex. Both *An. gambiae* s.s. and *An. coluzzii* were recorded. *An. coluzzii*, was the most abundant species identified representing 91.1% of the total whereas *An. gambiae* s.s. represented 8.9% of the total. Both species were found in sympatry in 55 out of 244 (22.5%) breeding habitats. *An. gambiae* s.s. larvae were collected alone in 2/244 breeding sites (0.8%). *An. coluzzii* was recorded alone in 76.6% (187/244) breeding habitats. Both species were present in breeding sites in the majority of districts of Yaoundé (Fig. 7).

### Multivariate regression analysis to assess factors affecting *An. gambiae* s.s. and *An. coluzzi* distribution in breeding sites

**Fig. 8:**
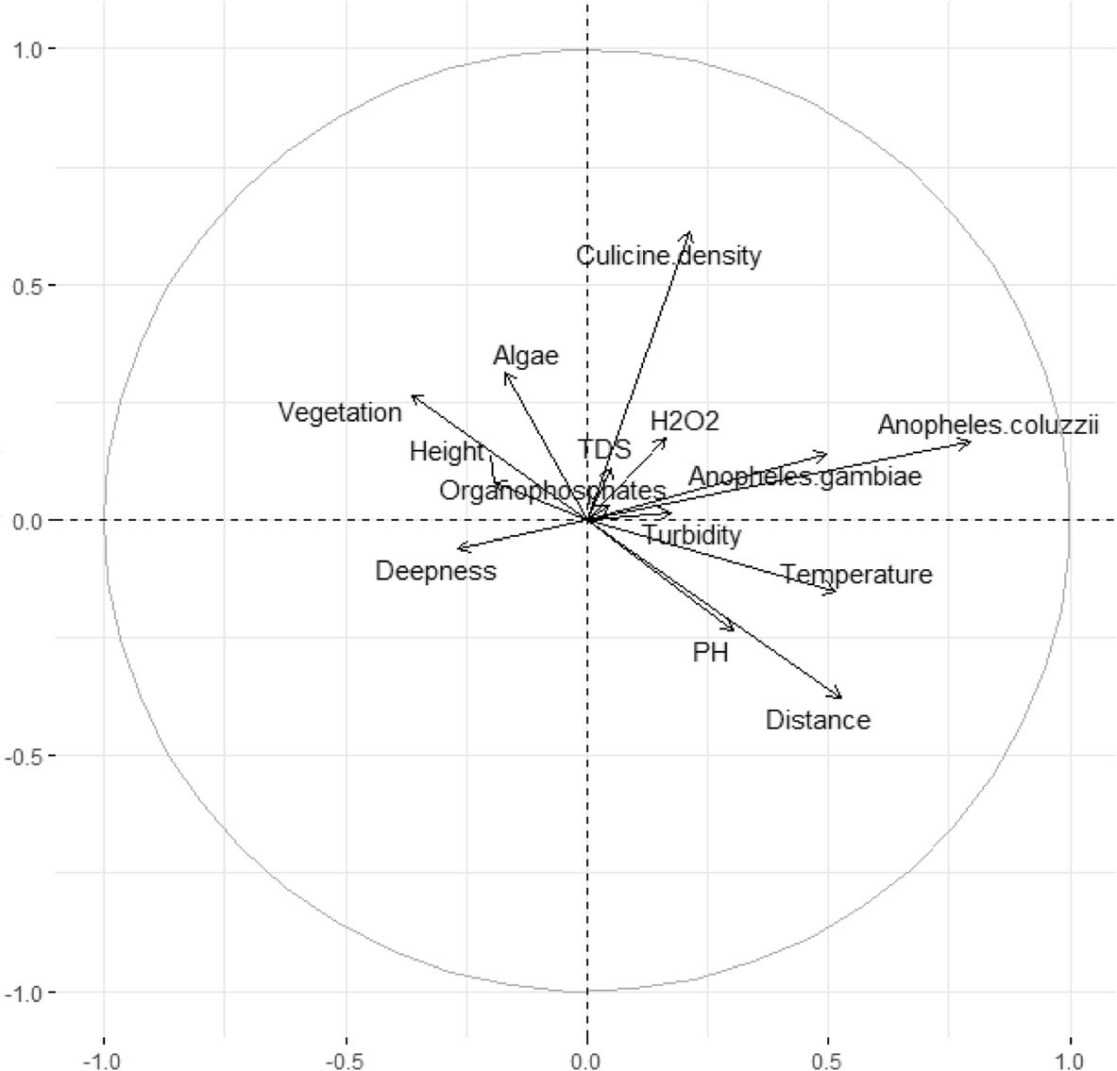
Multiple component analysis showing relationship between *An. gambiae s.s.*, *An. coluzzi* presence and some physical characteristics of breeding sites in Yaoundé

A multivariate analysis was used to assess the relationship between the presence of *An. gambiae* s.l. larvae and some environmental variables. A poor correlation was recorded with the first two axes of the diagram accounting for 15.5% of the total variance (Fig. 8). The first axis defined a gradient with *An. coluzzii* presence, anopheline density, distance to the nearest house, temperature, *An. gambiae* s.s. presence and immediate environment. The second environmental gradient was essentially associated with *Culex* presence, culicine density, immediate environment, anopheline density and distance to the nearest house. A positive and significant correlation was recorded between *An. gambiae* s.s. and *An. coluzzii* larvae distribution (*r* = 0.4; *P* <  0.0001). The temperature and pH were positively correlated to the distribution of both *An. gambiae* s.s. and *An. coluzzii* larvae (*r* > 0.13).

## Discussion

The study objective was to assess factors influencing the spatio-temporal distribution of *Anopheles gambiae* s.l. larvae in the city of Yaoundé. *An. gambiae* s.l. larvae were found to colonize a large number of habitats including temporary water collections, permanent sites and artificial habitats. This was in accordance with the high adaptation capacity of the species in urban settings [22, 48–50]. *An. gambiae* s.s. and *An. coluzzii* were recorded in sympatry in a high number of unpolluted (organic pollution) breeding sites. Yet *Anopheles coluzzii* was largely predominant with the densities outpassing by far those of *An. gambiae* s.s. This was in line with previous findings [20, 51, 52] supporting the high adaptation capacity of *An. coluzzii* to the urban environment. A certain number of physico-chemical parameters were found to affect the distribution of *An. gambiae* s.l. larvae in breeding sites. These included conductivity, turbidity, organophosphates and temperature which were in high concentration in breeding sites with anopheline larvae. Organic pollution was reported as a major factor affecting the distribution of both species. Indeed, *An. coluzzii* was reported to be more adapted to the presence of organic pollutants, whereas *An. gambiae* s.s. was not [19, 53, 54]. It is rather not known whether the reduction of organic pollutants in urban settings could favor recolonization of urban habitats by *An. gambiae* s.s. and this deserves further investigations.

Seasonal variations of aquatic habitats containing anopheline larvae were registered. March–April was the period when high larval density was recorded. Rainfall appears as a major factor influencing the distribution of anopheline larvae in breeding sites. It is rather possible that current climatic changes are affecting mosquito distribution and vector borne diseases epidemiology [55, 56]. In Cameroon since the late 1990s to date, an increase in temperature of + 0.4 °C compared to the period 1961–1990 and a reduction in rainfall ranging from − 10% to − 20% have been reported [57]. The density of larvae in breeding sites was highly variable and not dependent on the availability of aquatic habitats. The following could point to the influence of several factors such as environmental changes, the presence of pollutants in breeding sites, the availability of food resources or the presence of predators. The majority of breeding sites with anopheline larvae were temporary sites of less than 1 m, with no vegetation or algae, no predators, with a depth of less than 1 m. In Yaoundé in addition to temporary sites several permanent sites such as rivers edges, lacs, marshland exploited for agriculture are present all year long and contribute to maintain the presence of anopheline larvae in lowland areas. Although permanent sites were also recorded to harbor anopheline larvae, they were found to yield less larvae compare to temporary sites. This could result from the fact that, these sites are home to a wide range of invertebrate predators and competitors which could likely reduce mosquito larval density [58]. Several insects including odonata, toxorhynchitinae and culicine larvae (*Culex tigripes*) are known to be good predators of anopheline larvae and could influence the distribution and abundance of larvae in breeding habitats [59, 60]. Predators are known to reduce mosquito abundance by direct predation of early instars, they could also prevent females to oviposite in breeding sites or could affect mosquito population through competition for food resources [59, 61–65].

During the dry season the practice of agriculture in lowland areas in many districts over large surfaces was found to contribute to the distribution and maintenance of anopheline larvae presence. The clearing of vegetation and the creation of wells or irrigation canals for agriculture were found to be favorable habitats for mosquitoes. Also the practice of agriculture by increasing sedimentation due to erosion or direct elimination of vegetation or rubbish in the river bed which can slow or block streams and decrease the water depth could create shallow waters ideal for mosquito breeding [66]. In Yaoundé there is an extension of urban agricultural practices in a high number of sites. The contribution of urban agriculture to anopheline burden and malaria transmission in the city of Yaoundé has not received so far enough attention as elsewhere [67–71] and could be key for the control and elimination of malaria in the city of Yaoundé. Also because several pesticides are frequently used for controlling pest in these settings, these compounds by exerting a high selection pressure on mosquito populations contribute to the rapid expansion of insecticide resistance in the urban environment and across the country [22, 71–73] and deserve further consideration.

Several other activities closely associated to unplanned urbanisation were found to create suitable breeding habitats for mosquitoes. This included construction of houses in lowland areas or public work activities within the city, stagnant water bodies resulting from pipe leaking, car washing activities and the absence of drainage system for stagnant water bodies [74, 75]. Similar observations have been made by previous studies in different epidemiological settings [75–78]. The study also highlighted the need for an integrated approach for controlling malaria transmission in the city of Yaoundé.

The use of GIS software for mapping breeding habitats distribution in the city of Yaoundé provided important information that could be used for the management and supervision of larval control activities. This mapping showed rapid modification of the urban landscape with intense deforestation taking place around the city. GIS mapping contribution has been instrumental during the last decade in addressing issues related to malaria vector control in various setting across Africa [34, 79, 80]. The combination of field dataset and remotely sensed data provided a comprehensive picture of the changing landscape and mosquito distribution in the city of Yaoundé. From the analysis it appeared that the majority of anopheline breeding sites are found in lowland areas and that the pattern of mosquito distribution varied greatly between 2017 and 2018. The calculation of the I Moran index permitted to determine estimates that could be used to prioritize habitats or hotspots needing further attention. According to Tokarz and Novak [80], clusters displaying high outputs are indicative of multiple sites of high larval productivity and could certainly contribute highly to the *Anopheles* burden and are the utmost importance and need to serve as the top priority for control. The ability to identify and treat these clusters could significantly reduce the density of vector populations [81]. In this regard, weekly samplings rather than monthly collections as done in the present study, could be indicated to better capture the evolution of breeding sites productivity. From the study, it appeared that lowland areas where agriculture was practiced, were the most affected and need further attention. In the case of larval control trial as what is under preparation for the city of Yaoundé, focusing on such areas could be determinant for successful control and elimination of malaria in the urban environment.

Limitations from the study could come from the fact that, it was conducted in some districts, and this study could not capture all the variability in the city of Yaoundé. Also, field samplings were mainly conducted in standing water. Therefore, mosquito larvae collection would be underestimated the presence of other anopheline species such *An. funestus* in Yaoundé.

## Conclusions

The study provided a snapshot of the distribution of anopheline species before the implementation of a larval control trial in the city of Yaoundé. The study highlighted the fact that in addition to temporal sites in several hotspot areas mainly are associated to agricultural practices and contribute to perennial risk of malaria transmission in the city of Yaoundé. With the extension of the City, more efforts need to be undertaken in order to target hotspot areas such as marshlands exploited for agriculture and lakes, which contribute the most to malaria transmission. The use of larviciding or environmental management could be appropriate in the fight against malaria if these target hotspot areas where the use of LLINs could be insufficient for controlling malaria vectors.

## Supplementary information

**Additional file 1.** Multilingual abstracts in the five official working languages of the United Nations.

## Data Availability

Not applicable.

## Abbreviations

BI: Bulding Index
DEM: Digital Elevation Model
DNA: Desoxyribonucleic acid
GIS: Geographic information system
GPS: Global positionning system
LLINs: Long lasting insecticidal nets
NDVI: Normalised Difference Vegetation Index
NDWI: Normalized Difference Water Index
*OR*: Odd ratio
PCR: Polymerase chain reaction
TDS: Total dissolved solid
