# Supplementary material for: Spatial distribution of Anopheles gambiae sensu lato larvae in the urban environment of Yaoundé, Cameroon
Source: Infect Dis Poverty. 2019 Oct 9;8:84. doi: 10.1186/s40249-019-0597-6 (PMC6784347; doi:10.1186/s40249-019-0597-6)
Supplement: Supplementary file 1 — Additional file 1. Multilingual abstracts in the five official working languages of the United Nations. [file 40249_2019_597_MOESM1_ESM.pdf]

Translation of the abstract into the five official working languages of the United Nations

التوزع المكاني ليرقات الأنوفيلة الغامبية بالمعنى الواسع في البيئة الحضرية لياوندي بالكاميرون

لاندريه جاموكو جونكام، سليمان مونشيلي ندام، نيلي كالا شواكو، ستيلا مارييت نانا نجانغو، إدمون كوبيا، ناديج سونافوو شيانا، عبدو. طاليبو، كارمين ساندرا نغاجو، باتريسيا دومبيه بيليسيه، رولان بامو، جان كلود طوطو، تيموليون تشوينكام، شارل سينكلير وونجي، كريستوف أنطونيو نكونجيو

الملخص
السياق: من المعتقد أن يزيد التوسع العمراني السريع والعشوائي للمدن الإفريقية من خطر انتقال الملاريا في المناطق الحضرية. كان الهدف من هذه الدراسة هو تقييم العوامل المؤثرة في التوزع المكاني والزماني ليرقات الأنوفيلة الغامبية بالمعنى الواسع في مدينة ياوندي بالكاميرون.
الأساليب: تم فحص جميع المسطحات المائية مرة كل شهرين بحثا عن وجود يرقات البعوض من مارس 2017 إلى مايو 2018 في 32 مقاطعة في ياوندي. حيث تم تسجيل وتحليل الخصائص الفيزيائية والكيميائية بما في ذلك الحجم والعمق والتعكر ودرجة الحموضة ودرجة الحرارة والناقلية والكبريتات والفوسفات العضوي وبيروكسيد الهيدروجين ($_2O_2H$) والحديد والكالسيوم وفقا لوجود أو غياب يرقات الأنوفيلة. بينما تم استخدام صور فضائية عالية الدقة من الماسح الوظيفي المحسن لأقمار لاندسات وسنتينل من أجل رسم الخرائط لكل من المتغيرات الميدانية والبيئية. كما تم استخدام نماذج الانحدار اللوجستي ثنائي المتغير ومتعدد المتغيرات من أجل تحديد المتغيرات ذات الارتباط الوثيق بتوزع يرقات الأنوفيلة.
النتائج: تم فحص ما يبلغ مجموعه 18،696 من الموائل المائية ولم يتم العثور على يرقات الأنوفيلة إلا في 2،942 موقعا (15.7%). أظهر عدد كبير من المواقع المحتوية على يرقات الأنوفيلة (≤ 69%) وصول اليرقات إلى أطوار متأخرة (L3 وL4 وطور الشرنقة). حيث تم جمع العينات من يرقات بعوضة الأنوفيلة من عدد متنوع من مواقع التكاثر منها البرك (51.6%) وآثار الإطارات (12.9%) والآبار (11.7%) والمصارف (11.3%). أبرزت تحاليل الانحدار اللوجستي ثنائي المتغير ارتباط وجود يرقات الأنوفيلة بغياب المفترسين وانعدام الطحالب وعدم نمو النباتات وعدم تجاوز العمق لمتر واحد. بينما كانت معدلات كل من الناقلية والتعكر والفوسفات العضوي وبيروكسيد الهيدروجين ودرجة الحرارة مرتفعة إلى حد كبير في مواقع التكاثر المحتوية على يرقات الأنوفيلة مقارنة بمواقع التكاثر التي تخلو من هذه اليرقات ($0.1 > P$). وكان من بين الأنواع التي تم جمعها الأنوفيلة الكولوتسية (91.1%) والأنوفيلة الغامبية بالمعنى الدقيق (8.9%).
أظهرت خرائط نظام المعلومات الجغرافية توزعا غير متجانس لموائل تكاثر الأنوفيلة في مدينة ياوندي. كما أوضح تحليل الغطاء الأرضي تباينا كبيرا في المنظر العام لمدينة ياوندي.
الاستنتاجات: تؤكد البيانات تأقلم الأنوفيلة الغامبية بالمعنى الواسع مع المجال الحضري في مدينة ياوندي وتستدعي اتخاذ إجراءات عاجلة لتعزيز مكافحة ناقلات الملاريا.

Translated from English version into Arabic by Aghilas Mihoub, revised by Amine Abdoune, through

TRANSLATORS
WITHOUT BORDERS

喀麦隆雅温得城市环境中冈比亚按蚊幼虫的空间分布

Landre Djamouko-Djonkam, Souleman Mounchili-Ndam, Nelly Kala-Chouakeu, Stella Mariette Nana-Ndjangwo, Edmond Kopya, Nad ége Sonhafouo-Chiana, Abdou Talipouo, Carmene Sandra Ngadjeu, Patricia Doumbe-Belisse, Roland Bamou, Jean Claude Toto, Timol éon Tchuinkam, Charles Sinclair Wondji, Christophe Antonio-Nkondjio

摘要
引言：非洲城市的快速无计划的城市化增加了城市疟疾的传播风险。本研究的目的是评价影响喀麦隆雅温得市冈比亚按蚊幼虫时空分布的因素。
方法：2017 年 3 月～2018 年 5 月，每两个月对雅温得 32 个区的所有监测水体进行一次蚊幼虫检测。根据水体大小、深度、浊度、pH、温度、电导率、硫酸盐含量、有机磷含量、过氧化氢(H_2O_2)含量、电导率、铁和钙离子浓

度等理化特性结合按蚊幼虫孳生与否进行记录和分析。利用 landsat sentinel 增强成像的高分辨率卫星图像对野外和环境变量进行空间制图。采用双变量和多变量 logistic 回归模型来确定与按蚊幼虫分布密切相关的变量。
结果：共检查水生环境 18 696 处，其中含按蚊幼虫 2942 处(15.7%)。超过 69%的调查水体中的幼虫为高龄幼虫(L3、L4 和蛹)。孳生地类型包括水坑(51.6%)、轮胎印(12.9%)、水井(11.7%)和排水沟(11.3%)。双变量 logistic 回归分析结果提示按蚊幼虫的存在与无捕食者、无藻类、无植被和水体深度小于 1 m 有关。按蚊幼虫孳生地的电导率、浊度、有机磷、H_2O_2 和温度显著高于无幼虫的调查水体($P < 0.1$)。所采集的按蚊种类包括柯氏按蚊(91.1%)和冈比亚按蚊 (8.9%)。GIS 图像显示雅温得地区按蚊孳生地分布不均匀。土地覆盖物分析表明，雅温得城市环境具有高度的可变性。
结论：本研究结果证实了冈比亚按蚊对雅温得城市地区的适应性，并呼吁采取紧急行动改善疟疾媒介控制措施。

Translated from English version into Chinese by Peng Song, edited by Pin Yang

CHINESE CENTER FOR DISEASE CONTROL AND PREVENTION
NATIONAL INSTITUTE OF PARASITIC DISEASES

La répartition spatiale des larves *Anopheles gambiae* sensu lato dans l'environnement urbain de Yaoundé, Cameroon

Landre Djamouko-Djonkam, Souleman Mounchili-Ndam, Nelly Kala-Chouakeu, Stella Mariette Nana-Ndjangwo, Edmond Kopya, Nadège Sonhafouo-Chiana, Abdou Talipouo, Carmene Sandra Ngadjeu, Patricia Doumbe-Belisse, Roland Bamou, Jean Claude Toto, Timoléon Tchuinkam, Charles Sinclair Wondji, Christophe Antonio-Nkondjio

Résumé

Contexte: L'urbanisation rapide et imprévue des villes africaines peut augmenter le risque de transmission de la malaria urbaine. L'objectif de la présente étude était d'évaluer les facteurs qui influent la répartition spatio-temporelle des larves *Anopheles gambiae* sensu lato dans la ville de Yaoundé, Cameroon.
Les méthodes: Tous les plans d'eau ont été inspectés tous les deux mois pour détecter la présence de larves de moustiques de mars 2017 à mai 2018, dans 32 districts de Yaoundé. Des caractéristiques physico-chimiques incluent la taille, la profondeur, la turbidité, le pH, la température, la conductivité, les sulfates, les organophosphorés, le peroxyde d'hydrogène (H_2O_2), la conductivité, le fer et le calcium ont été enregistrés et analysés en fonction de la présence ou de l'absence de larves d'anophèles. Des images satellites haute résolution de landsat sentinel Enhanced Thematic Mapper é ont été utilisées pour la cartographie spatiale des variables de terrain et environnementales. Des modèles de régression logistique bivariés et multivariés ont été utilisés pour identifier les variables associées à la distribution des larves d'anophèles.
Les résultats: Un total de 18 696 habitats aquatiques ont été inspectés et seulement 2942 sites (15.7 %) contenaient des larves d'anophèles. Un grand nombre de sites contenant des larves d'anophèles (≥ 69%) ont présenté des larves (L3, L4 et pupes). Les larves de moustiques anophèles ont été échantillonnées de divers sites de reproduction, comme des flaques d'eau (51,6%), des empreintes de pneus (12,9%), des puits (11,7%) et des drains (11,3%). Les analyses de régression logistique bivariées associaient la présence de larves d'anophèles à l'absence de prédateurs, à l'absence d'algues, à l'absence de végétation et à une profondeur moins de 1 m. La conductivité, la turbidité, les organophosphorés, H_2O_2 et la température étaient significativement élevées dans les sites de reproduction avec des larves d'anophèles par rapport aux sites de reproduction sans ces larves (P 0,1). Les espèces anophèles recueillies incluent *An. coluzzii* (91,1%) et *An. gambiae* stricto sensu (8,9%). La cartographie SIG a révélé une répartition hétérogène des habitats de reproduction des anophèles dans la ville de Yaoundé. L'analyse de la couverture terrestre a révélé une grande variabilité du paysage de la ville de Yaoundé.

Les conclusions: Les données confirment l'adaptation de *An. gambiae* s.l. dans le domaine urbain de la ville de Yaoundé et demande des actions urgentes pour améliorer le contrôle des vecteurs de la malaria.

Translated from English version into French by Anna Cho, revised by Ahmad Dabaghzadeh, through

TRANSLATORS WITHOUT BORDERS

Территориальное распределение личинок малярийного камара *(Anopheles gambiae)* в городской среде Яунде, Камерун

Ландре Джамуко-Джонкам, Сулеман Мунчили Ндам, Нелли Кала-Чуакеу, Стелла Мариетт Нана-Нджангво, Эдмонд Копья, Надеж Сонхафуо-Киана, Абду Талипуо, Кармен Сандра Нгаджеу, Патриция Думбе-Белисс, Ролан Баму, Жан Клод Тото, Тимолеон Чуинкам, Чарльз Синклер Вонджи, Кристоф Антонио-Нконджио

Аннотация

Предпосылки исследования: Считается, что быстрая и незапланированная урбанизация африканских городов повышает риск передачи малярии в городах. Целью настоящего исследования было оценить факторы, влияющие на пространственно-временное распределение личинок *Anopheles gambiae* в городе Яунде, Камерун.

Методы: Два раза в месяц с марта 2017 года по май 2018 год проводилась проверка водных резервуаров на наличие личинок комаров в 32 районах города Яунде. В зависимости от наличия или отсутствия личинок малярийного комара проводился учет и анализ физико-химических характеристик воды, включая размер, глубину, плотность, кислотность, температуру, удельную электропроводимость, содержание сульфатов, органических фосфатов, перекиси водорода (H_2O_2), железа и кальция. Спутниковые снимки высокого разрешения с усовершенствованного тематического картографа Landsat Sentinel использовались для пространственного картирования как полевых переменных , так и параметров окружающей среды. Двумерные и многомерные модели логистической регрессии были использованы для определения переменных, тесно связанных с распространением личинок малярийного комара.

Результаты: Всего было проверено 18 696 водных сред обитания, и только в 2942 участках (15,7%) содержались личинки малярийного комара. Большое количество участков с личинками малярийного комара (≥ 69%) представлено личинками поздней стадии (L3, L4 и куколки). Отбор личинок малярийного комара проводился в различных местах размножения, включая лужи (51,6%), отпечатки шин (12,9%), колодцы (11,7%) и стоки (11,3%). Двусторонний логистический регрессионный анализ связал наличие личинок малярийного комара с отсутствием хищников, водорослей, растительности и глубиной менее 1 м. Электропроводимость, плотность, органические фосфаты, H_2O_2 и температура были значительно выше в местах размножения личинок малярийного комара, чем в местах размножения без этих личинок (*P* 0.1). Собранные виды малярийного комара включали *An. coluzzii* (91,1%) и *An. gambiae* sensu stricto (8,9%). Картографирование ГИС показало неоднородное распределение мест обитания и размножения малярийного комара в городе Яунде. Анализ земляного покрова показал высокую изменчивость ландшафта города Яунде.

Выводы: Данные подтверждают адаптацию *An. gambiae* s.l. в городской среде города Яунде и призывают к принятию срочных мер по усилению борьбы с переносчиками малярии.

Translated from English version into Russian by Tatiana Karymshakova, revised by Gunel Huseynbayova, through

Distribución espacial de las larvas del *Anopheles gambiae* sensu lato en el entorno urbano de Yaoundé, Camerún

Landre Djamouko-Djonkam, Souleman Mounchili-Ndam, Nelly Kala-Chouakeu, Stella Mariette Nana-Ndjangwo, Edmond Kopya, Nadége Sonhafouo-Chiana, Abdou Talipouo, Carmene Sandra Ngadjeu, Patricia Doumbe-Belisse, Roland Bamou, Jean Claude Toto, Timoléon Tchuinkam, Charles Sinclair Wondji, Christophe Antonio-Nkondjio

Resumen

Antecedentes: Se considera que la urbanización rápida y no planificada de las ciudades africanas aumenta el riesgo de transmisión urbana del paludismo. El objetivo del presente estudio fue evaluar los factores que influyen en la distribución espaciotemporal de las larvas del *Anopheles gambiae* sensu lato en la ciudad de Yaundé, Camerún.

Métodos: Una vez cada dos meses, se examinaron todas las masas de agua para detectar la presencia de larvas de mosquitos, desde marzo de 2017 a mayo de 2018, en 32 distritos de Yaoundé. Se registraron y analizaron, de acuerdo con la presencia o ausencia de larvas de anofelina, características fisicoquímicas que incluyen tamaño, profundidad, turbidez, pH, temperatura, conductividad, sulfatos, organofosforados, peróxido de hidrógeno (H_2O_2), conductividad, hierro y calcio. Se utilizaron imágenes de satélite de alta resolución del cartógrafo temático perfeccionado del landsat sentinel para la cartografía espacial de variables de campo y ambientales. Se utilizaron modelos de regresión logística bivariable y multivariable para identificar variables estrechamente asociadas con la distribución de larvas de anofelina.

Resultados: Se controlaron un total de 18 696 hábitats acuáticos y sólo 2942 sitios (15,7%) contenían larvas de anofelina. Un alto número de sitios con larvas de anofelina (≥ 69%) presentaron larvas tardías en fase terminal (L3, L4 y pupa). Se tomaron muestras de larvas de mosquito anofelina de una variedad de sitios de reproducción, incluidos charcos (51,6%), huellas de neumáticos (12,9%), pozos (11,7%) y drenajes (11,3%). Los análisis de regresión logística bivariable asociaron a la presencia de larvas anofelinas con la ausencia de depredadores, algas y vegetación, y con una profundidad de menos de 1 m. La conductividad, la turbidez, los organofosfatos, el H_2O_2 y la temperatura fueron significativamente más altos en los criaderos con larvas anofelinas que en los criaderos sin ellas ($P < 0{,}1$). Las especies de anopheline obtenidas incluyeron *An. coluzzii* (91,1%) y *An. gambiae* sensu stricto (8,9%). El mapeo SIG indicó una distribución heterogénea de hábitats de reproducción anofelina en la ciudad de Yaundé. El análisis de la cobertura del suelo indicó una gran variabilidad de paisaje de la ciudad de Yaundé.

Conclusiones: Los datos confirman la adaptación del *An. gambiae* s.l. al dominio urbano en la ciudad de Yaoundé y llaman a la adopción urgente de medidas para mejorar el control de los vectores del paludismo.

Translated from English version into Spanish by María Luz Puerta, revised by Maria Paula Gorgone, through
